# Laparoscopic vs. Open Approach in Emergent Inguinal Hernia: Our Experience and Review of Literature

**DOI:** 10.3389/jaws.2023.11242

**Published:** 2023-06-16

**Authors:** Francisco Moreno-Suero, Luis Tallon-Aguilar, José Tinoco-González, Alejandro Sánchez-Arteaga, Juan Manuel Suárez-Grau, Miriam Alvarez-Aguilera, Salvador Morales-Conde, Javier Padillo-Ruiz

**Affiliations:** Virgen del Rocío University Hospital, Seville, Spain

**Keywords:** inguinal hernia, hernia repair, incarcerated, laparoscopic surgery, emergency surgery

## Abstract

There is currently no consensus or homogeneous recommendation about the role of the laparoscopic approach in emergent inguinal hernia surgery. The aim of this manuscript is showing our experience and results of laparoscopic approach for emergent groin hernia repair comparing with open approach. A retrospective review of a prospectively maintained database between January 2011 and December 2021 of acute incarcerated groin hernia that were operated at Virgen del Rocio University Hospital. In this period, they were identified 463 patients with groin hernia that required an emergency repair. 454 patients underwent open surgery (group 1) and 36 patients underwent laparoscopic approach (TAPP procedure) (group 2). Median length stay was 1 day in lap group and 2 days in open approach. Reintervention was necessary in 20 cases (4.40%) from group 1 and one (2.27%) from group 2. In laparoscopic approach, no mortality was described but in open approach, 10 patients (2.20%) died. Globally, 58 cases (12.77%) from group 1 and six patients (16.66%) from group 2 presented any complication. Wound infection was higher in group of open repairs (5.94% vs. 2.77%). Non-surgical complications were higher in open approach (19 vs. 0). There is no statistical significance in any of these items. Laparoscopic approach is a safe, feasible and effective therapeutic option for the treatment of incarcerated groin hernia that require emergency surgery, but prospective and randomized comparative studies are needed to establish the best approach.

## Introduction

Inguinal hernia is by far the most common abdominal wall pathology, with an occurrence of up to 75% in some series, and its repair is one of the main surgical procedures performed by General Surgeons worldwide [1–3]. However, inguinal hernia repair technique and approach are deeply conditioned by the urgency of the intervention due to the hernia’s incarceration and a subsequent bowel obstruction.

Following postoperative adhesions, incarcerated groin hernia is the second most common cause of acute bowel obstruction. Moreover, 15% of patients undergoing emergency surgery for incarcerated groin hernia require intestinal resection, associated with a non-despicable morbidity and mortality rate in comparison to non-obstructive elective hernia repair [4, 5].

Different techniques and approaches for inguinal hernia repair have been introduced parallel to surgery development in recent decades. In this context, the introduction of minimally invasive approaches in elective inguinal hernia repair has already demonstrated many advantages: a faster return to daily activities, less postoperative pain and analgesic consumption, and lower rate of wound infection when comparing to open approaches [6–9]. Nevertheless, the application of laparoscopic approaches to emergent repairs is still under discussion [4, 9, 10]. There are a few publications defending the advantages of laparoscopic repairs but most of them are based on low evidence studies (retrospective case series or retrospective cohort studies). To our knowledge, only one recent systematic review has provided strong information on these minimally invasive advantages in emergent inguinal hernia repair, reporting a shorter surgical time, shorter hospital stays, and lower surgical site infection rate with a similar recurrence rate [9].

This scarcity of studies resulted in the latest International Guidelines on groin hernia management highlighting the lack of evidence needed to recommend a standard approach for emergent inguinal hernia repair [4, 11–14]. Thus, there is currently no consensus about the role of the laparoscopic approach in emergent inguinal hernia surgery.

In this sense, the aim of this manuscript is to throw some light on this important issue by sharing our experience and results with laparoscopic approaches for emergent groin hernia repair compared to open approaches.

## Materials and Methods

### Study Design

A retrospective analysis of a prospectively maintained database of a reference center was carried out, including patients who were operated on due to acute incarcerated groin hernia from 1st January 2011 to 31st December 2021.

### Patients

Inclusion criteria were: Patients older than 18 years, with uni or bilateral acute incarcerated groin hernia requiring emergent surgery (any technique or approach).

A total of 490 patients met the inclusion criteria and were included in the study in two separate groups depending on the surgical approach: Open approach (Group 1); 454 patients, and laparoscopic approach—TAPP technique—(Group 2); 36 patients.

### Surgical Procedure

All procedures were performed by the experienced surgeons included in the Abdominal Wall Reconstruction Department (M-S, S-A, T-G, S-G and T-A) following national and international hernia management guidelines [13, 14] and tailored for each patient’s characteristics. Postoperative care was provided according to our hospital protocol matching Enhanced Recovery After Surgery (ERAS) protocol.

### Variables

Baseline characteristics, hernia type, surgical procedure, hospital stay, ICU admission, reintervention, hospital readmission, perioperative complications, and mortality.

### Statistical Analysis

Statistical analysis was carried out using SPSS version 25.0 for Windows (SPSS Inc. Chicago IL). A descriptive analysis of the different frequencies and distribution of observed variables has been performed. Subsequently, we verified whether the quantitative variables followed a normal distribution via the Shapiro-Wilks normality test. The association between variables with parametric and non-parametric methods were evaluated according to correspondence. Some of the bivariate analyses that were performed were the Chi-Square test and the Student’s t-test. In all statistical analyses, the significance level was set at *α* = 0.05.

## Results

No statistical difference was found in terms of baseline characteristics in both groups (Table 1). The mean age was 69.15 years (SD ± 15.96) in Group 1 and 65.2 years (SD ± 14.47) in Group 2 (*p* = 0.167). Regarding gender, there was a majority of male patients in both groups, 272 (59.91%) in Group 1 and 24 (66.67%) in Group 2 (*p* = 0.419). Similarly, bowel resection was equally distributed in both groups, 57 (12.55%) patients underwent bowel resection in Group 1, and 4 (11.1%) in Group 2 (*p* = 0.790). Four conversions were described in the laparoscopic approach group.

**TABLE 1:** Results Open vs. laparoscopic approach.

	Open approach (Group 1)	Laparoscopic approach (Group 2)	P
N	454	36	
Male gender	272 (59.91%)	24 (66.67%)	0.419
Age (average, SD)	69.15 (SD ± 15.96)	65.2 (SD ± 14.47)	0.167
Hospital stay (average, SD)	4.87 (SD ± 11.5)	2.88 (SD ± 4.16)	0.329
Hospital stay (median, range)	2 (0–184)	1 (0–36)	0.329
Bowel resection	57 (12.55%)	4 (11.1%)	0.790
Anastomosis leak	2/57 (3.51%)	1/4 (25%)	0.085
Wound infection	27 (5.94%)	1 (2.77%)	0.429
Intraabdominal abscess	4 (0.8%)	1 (2.77%)	0.277
Bleeding	1 (0.22%)	1 (2.77%)	0.834
Evisceration	3 (0.66%)	0 (0%)	0.624
Reintervention	20 (4.40%)	1 (2.77%)	0.641
Hospital readmission	7 (1.54%)	2 (5.55%)	0.085
Catheter sepsis	2 (0.44%)	0 (0%)	0.690
Respiratory infection	10 (2.20%)	0 (0%)	0.368
Cardiac complication	7 (1.76%)	0 (0%)	0.453
ICU admission	20 (4.40%)	1 (2.77%)	0.641
Death	10 (2.20%)	0 (0%)	0.789
Complication (global)	58 (12.77%)	6 (16.66%)	0.508

In terms of postoperative results, results were also quite parallel in both groups. In total, 58 (12.77%) patients from Group 1, and 6 (16.66%) from Group 2 presented with any sort of complication (*p* = 0.508). Surgical Site infection was higher in the Open Approach group but with no statistical significance [27 (5.94%) vs. 1 (2.77%), *p* = 0.429]. Only one case of postoperative bleeding was observed in each group [1 (0.22%) vs. 1 (2.27%), *p* = 0.834], and a postoperative intraabdominal abscess was developed in 4 (0.8%) patients in group 1, against 1 (2.77%) in Group 2 (*p* = 0.277). Furthermore, there were no statistical differences in systemic complications in both groups, although Group 2 patients suffered from 2 instances of catheter-related sepsis (0.44%), 10 respiratory infections (2.20%), and 7 cardiac complication (1.76%) while none of these complications appeared in the laparoscopic group.

Median length stay was 2 days (0–184) in Group 1 and 1 day (0–34) in Group 2 (*p* = 0.329). Average hospital stay was 4.87 days (SD ± 11.5) and 2.88 days (SD ± 4.16) days, respectively (*p* = 0.329). ICU admission was required for 20 patients (4.40%) from Group 1, and 1 (2.77%) patient in Group 2 (*p* = 0.641). Reintervention was necessary in 20 cases (4.40%) from Group 1, and 1 case (2.27%) from Group 2 (*p* = 0.641).

Despite no statistical difference being found, 10 patients (2.20%) died in the Open Approach Group, whereas no mortality was observed in the Laparoscopic Approach Group, (*p* = 0.789).

## Discussion

A minimally invasive approach for elective groin hernia repair has been well documented in literature and is already implemented in routine clinical practice. However, this approach is still controversial for the management of incarcerated or strangulated inguinal hernia in emergency surgery. The main problems reported by surgeons who oppose its standardization are the difficulty of reducing the hernia’s sac and the risk of iatrogenic injuries [2–4, 15].

The first laparoscopic repair for incarcerated groin hernia was reported in 1993 [16]. Since then, several publications have tried to throw some light on this topic. In 1996, Ishihara et al [17] reported a series of cases using the TAPP technique for the reduction of incarcerated hernias assessing bowel viability intra-operatively, only one patient’s intervention ended up needing surgical conversion into an open laparotomy approach. A few years later, in 2004, Ferzli et al [18], described their results in 11 patients with acute hernias operated via TEP approach; of them, three patients needed an eventual conversion into open approach, two patients presented with any sort of postoperative complication, and one of them needed bowel resection. The mean hospital stay was 5.4 days. Since the TEP technique does not allow for assessment of the intra-abdominal cavity and full bowel viability, the TAPP technique could seem safer in emergency groin hernia repairs.

More recently, in 2009, Deeba et al [4] published their study focused on the minimally invasive treatment of acutely incarcerated inguinal hernia, including 328 patients. Their sample’s results were: 34 complications (10.36%), 25 of which were reported as minor, six conversions into Open Approach, an average operating time of 61.3 min (SD ± 12.3), and an average hospital stay of 3.8 days (SD ± 1.2). Thus, quite similar to our sample’s results.

As previously mentioned, to our knowledge, the highest quality study currently published is the systematic review and meta-analysis of Sartori et al. [9]. Fifteen articles were included comparing minimally invasive vs. open approaches in emergency groin hernia. Their results were better for the laparoscopic group, describing a shorter mean operative time and hospital stay, lower postoperative complications of 16 (9.8%) vs. 57 (24.3%), and especially a lower rate of wound infection (2.77% vs. 5.94%). All of the above are consistent with our sample’s results, even though we could not find a statistically significant difference, probably due to our Group 2 size limitation.

On the other hand, for Sartori et al., the two approaches showed equivalent results in terms of postoperative hematoma. Unfortunately, another limitation to our study could be that our historical database was not designed to assess postoperative hematoma, only bleeding that led to intervention, which was similar in both groups [9].

We consider important to keep in mind that laparoscopic approaches also have some handicaps and limitations, absolute contraindications could be hemodynamic instability and contraindication to general anesthesia, while some relative contraindications could be technical limitations to perform the surgery (very large hernias or need for bowel resection). Furthermore, we think that minimally invasive approaches for emergent inguinal hernia should only be performed by groups with experience in elective surgery, presenting a longer and more complex learning curve. Training plays a key role in this regard.

Even if recent evidence leads to the presumption that minimally invasive approaches could have better results than open approach, no prospective or randomized studies have been designed yet and patients in previous reviews seem biased by the selection of those in better clinical condition.

Because of this, in 2018, the HerniaSurge Group Guidelines stated that due to the lack of decisive evidence of superiority of one approach over another, in the case of acute incarcerated inguinal hernia, a tailored approach is suggested [13]. Parallel to that, the World Society of Emergency Surgery (WSES) Guidelines considered laparoscopic approaches as a useful tool for emergency abdominal wall hernia repair, especially due to its possibility to assess bowel viability even after spontaneous reduction (grade 2B recommendation).

In the case of incarcerated or spontaneously reduced inguinal hernia, the laparoscopic approach allows us to check the sac contents, and makes it possible to perform an intraoperative fluorescence angiography with indocyanine green in case of doubt to check bowel perfusion [19].

On the whole, the scarcity, heterogeneity, and limitations of the published studies to date leave us with no clear evidence on this topic. For this reason, our group is leading a prospective randomized multicenter clinical trial comparing open versus laparoscopic approach in emergency inguinal hernia repair, aiming to have a high evidence assessment of postoperative results in both approaches. The study is called INGURLAP and was awarded the 2021 EHS research grant. It is up and running, with approximately 60% of the sample already recruited.

## Conclusion

Laparoscopic approach is a safe, feasible, and effective therapeutic option for emergent incarcerated groin hernia repair.

A minimally invasive laparoscopic approach seems to have many advantages when compared to open approaches both during surgery (bowel viability assessment) and in postoperative results.

Prospective and randomized comparative studies are needed to establish the best approach for emergency groin hernia repair. We hope that INGURLAP study will help to improve the available evidence and highlight the role of a laparoscopic approach in the treatment of emergency groin hernia repair.
